# Introduction to the database “efficacy of different treatment modalities for lower respiratory tract infections in children”

**DOI:** 10.1002/pdi3.79

**Published:** 2024-07-02

**Authors:** Yu Deng, Bing Hu, Yingxue Zou, Zhengxiu Luo, Guangli Zhang, Jinhai Ma, Changshan Liu, Xiaoyan Dong, Huifen Zi, Chuangli Hao, Rongjun Lin, Xiangrong Zheng, Bingfei Li, Fenhua Chen, Mei Fang, Weimin Tian, Zhiqiang Zhuo, Deyu Zhao, Zhimin Chen, Yuejie Zheng, Jingyang Zheng, Yong Yin, Qiuyu Tang, Liqun Wu, Li Gu, Jinzhun Wu, Liyi He, Tao Ai, Ning Wang, Minjun Zhang, Hailin Zhang, Hanmin Liu, Youxiang Zhang, Jianguo Hong, Zhiying Han, Yunbo Mo, Hongmei Qiao, Zhiliang Tian, Zengni Li, Quan Lu, Enmei Liu

**Affiliations:** ^1^ Department of Respiratory Center Children's Hospital of Chongqing Medical University National Clinical Research Center for Child Health and Disorders Ministry of Education Key Laboratory of Child Development and Disorders Chongqing Key Laboratory of Child Infection and Immunity Chongqing China; ^2^ Yichun People's Hospital Yichun Jiangxi China; ^3^ Tianjin Children's Hospital Tianjin China; ^4^ General Hospital of Ningxia Medical University Yinchuan Ningxia China; ^5^ The Second Hospital of Tianjin Medical University Tianjin China; ^6^ Shanghai Children’s Hospital School of Medicine, Shanghai Jiao Tong University Shanghai China; ^7^ Baotou City Central Hospital Baotou, Inner Mongolia China; ^8^ Children's Hospital of Soochow University Suzhou Jiangsu China; ^9^ The Affiliated Hospital of Qingdao University Qingdao Shandong China; ^10^ Xiangya Hospital Central South University Changsha Hunan China; ^11^ Ganzhou Women and Children's Health Care Hospital Guangzhou Guangdong China; ^12^ The Third Affiliated Hospital Sun Yat‐sen University Guangzhou Guangdong China; ^13^ Xiamen Medical College Affiliated Haicang Hospital Xiamen Fujian China; ^14^ Zhongshan Hospital Xiamen University Xiamen Fujian China; ^15^ Xiamen Children's Hospital Xiamen Fujian China; ^16^ Children's Hospital of Nanjing Medical University Nanjing Jiangsu China; ^17^ Children's Hospital of Zhejiang University School of Medicine Hangzhou Zhejiang China; ^18^ Shenzhen Children's Hospital Shenzhen Guangdong China; ^19^ Quanzhou Children's Hospital of Fujian Province Quanzhou Fujian China; ^20^ Shanghai Children's Medical Center Shanghai China; ^21^ The First Affiliated Hospital of Fujian Medical University Fuzhou Fujian China; ^22^ Dongfang Hospital Beijing University of Chinese Medicine Beijing China; ^23^ Shanghai Tenth People's Hospital Shanghai China; ^24^ The First Affiliated Hospital of Xiamen University Xiamen Fujian China; ^25^ Women and Children's Hospital School of Medicine, Xiamen University Xiamen China; ^26^ The First People's Hospital of Foshan Foshan Guangdong China; ^27^ Chengdu Women's and Children's Central Hospital Chengdu Sichuan China; ^28^ Xi'an Children's Hospital Xi'an Shanxi China; ^29^ Fujian Provincial Maternal and Child Health Care Hospital Fuzhou Fujian China; ^30^ The Second Affiliated Hospital of Wenzhou Medical University Wenzhou Zhejiang China; ^31^ West China Second University Hospital Sichuan University Chengdu Sichuan China; ^32^ Guangzhou First People's Hospital Guangzhou Guangdong China; ^33^ Shanghai General Hospital Shanghai China; ^34^ Children's Hospital of Shanxi Taiyuan Shanxi China; ^35^ Chongqing University Three Gorges Hospital Chongqing China; ^36^ The First Hospital of Jilin University Changchun Jilin China; ^37^ The Second Affiliated Hospital of Harbin Medical University Harbin Heilongjiang China

**Keywords:** children, database, different treatment modalities, efficacy, lower respiratory tract infection

## Abstract

This study aimed to establish a registry database of different treatment modalities for lower respiratory tract infections (LRTIs) in Chinese children, thereby filling gaps in knowledge on clinical characteristics, treatment modalities, and rational drug use in relation to LRTIs in Chinese children, and providing large amounts of continuous, complete, scientific, and objective clinical data, and an information exchange platform. Multicenter data from these children's clinical visits were collected, pooled, and analyzed using medical informatics and statistical techniques to explore their potential value. The database was preliminarily established and a real‐world study cohort was constructed based on a total of 4805 patients registered in this database. Pneumonia was identified as the most common type of LRTIs (72.44%), followed by acute bronchitis (20.71%). The mean age of the enrolled children with LRTIs was 3.26 ± 2.84 years, and boys accounted for 59.21% of the samples. Among the enrolled children, pneumonia and acute bronchitis had the highest incidence in children aged 1–3 years (27.44%) and those aged 3–6 years (34.16%), respectively. In this national, multicenter, observational database of LRTIs in children, the real‐world characteristics and treatment modalities for LRTIs in Chinese children are elucidated. This database will help improve the research efficiency of clinicians and facilitate the exploration of underlying clinical patterns in real‐world medical big data.

## Graphical abstract

This article introduces a national, multicenter, prospective, non‐interventional, observational registry study, which established a comprehensive and sustainable database of lower respiratory tract infection in children. This database will help improve the scientific research efficiency of clinicians and facilitate the exploration of underlying clinical patterns within the realm of real‐world medical big data.

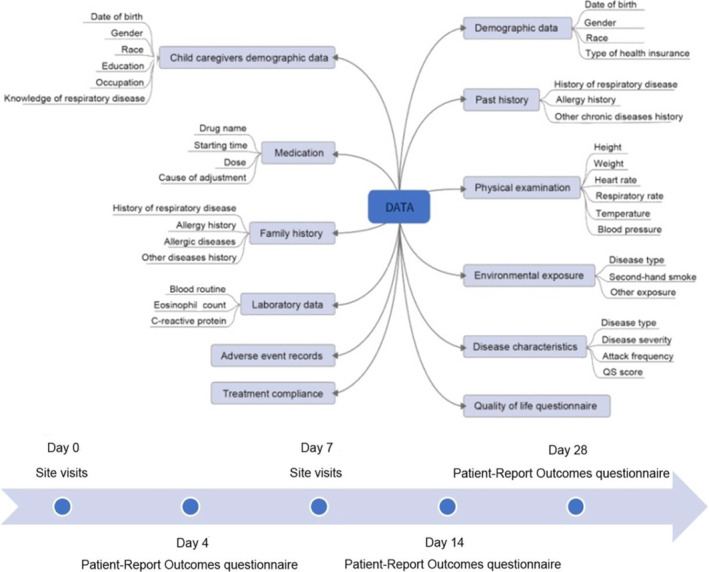

## 1 INTRODUCTION

In China, lower respiratory tract infections (LRTIs) have the highest incidence rate among all pediatric diseases, constituting 39.0%–65.5% of pediatric outpatients and 24.5%–65.2% of pediatric inpatients.^1^, ^2^ According to the 2017 Global Burden of Disease report, the total burden of LRTIs diseases in China was 1,858,788.35 persons/year in children under 5 years of age and 147,963.22 persons/year in children aged 5–14 years, with a mortality rate of 26.32 cases per 100,000 persons among children under 5 years of age.^3^, ^4^ According to the statistics of the World Health Organization, approximately 156 million children worldwide contract pneumonia annually, of which 151 million cases occur in developing countries, including 21 million in China, second only to India.^5^ Hence, it is imperative to strengthen preventive and therapeutic measures for LRTIs in children.

At present, there has been no large‐scale, real‐world study on the clinical characteristics, treatment modalities, and rational drug use for LRTIs in Chinese children. Thus, we designed this national, multicenter, non‐interventional, real‐world study of LRTIs in Chinese children to provide a valuable reference for rational drug use in clinical practice.

Accordingly, we designed and conducted this registry database study on the efficacy of different treatment modalities for LRTIs in children under the guidance of Professor Enmei Liu from the Children's Hospital of Chongqing Medical University and Professor Quan Lu from Children's Hospital of Shanghai. The study data were collected from 36 hospitals across the nation, including tertiary general hospitals, maternal and child healthcare centers, and specialized children's hospitals. The objective of this study was to elucidate and introduce the database's establishment process, data collection, data processing, and current status.

## 2 METHODS

### 2.1 Data source and collection period of the database

**FIGURE 1:**
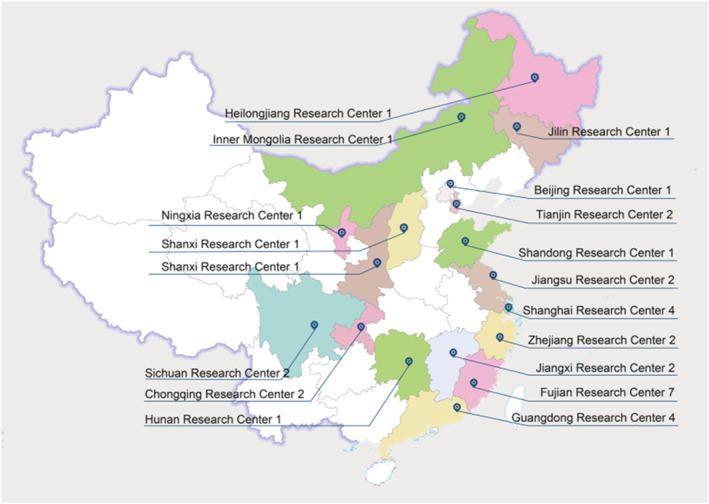
Distribution of specific study sites.

Date in the database were collected by pediatric respiratory specialists with prescription rights in inpatient and outpatient departments of 36 hospitals from 12 provinces in China, including 4 municipalities directly under the Central Government and 2 autonomous regions. The specific study sites are shown in Table 1 and Figure 1. Clinical data and related information were collected from 4805 pediatric patients since the enrollment of the first pediatric patient on May 23, 2018 and continued until July 31, 2019.

**TABLE 1:** Research centers and provinces/municipality/autonomous regions.

Province/municipality	Number of hospitals	Hospital name
Tianjin	2	Tianjin Children's Hospital
		The Second Hospital of Tianjin Medical University
Beijing	1	Dongfang Hospital Beijing University of Chinese Medicine
Inner Mongolia	1	Baotou City Central Hospital
Shanxi	1	Children's Hospital of Shanxi
Shanghai	4	Shanghai Children’s Hospital, School of Medicine, Shanghai Jiao Tong University
		Shanghai General Hospital
		Shanghai Tenth People's Hospital
		Shanghai Children's Medical Center
Jiangxi	2	Yichun People's Hospital
		Ganzhou Women and Children's Health Care Hospital
Shandong	1	The Affiliated Hospital of Qingdao University
Jiangsu	2	Children's Hospital of Soochow University
		Children's Hospital of Nanjing Medical University
Zhejiang	2	The Second Affiliated Hospital of Wenzhou Medical University
		The Children's Hospital of Zhejiang University School of Medicine
Fujian	7	The First Affiliated Hospital of Xiamen University
		Xiamen Children's Hospital
		Fujian Provincial Maternal and Child Health Hospital
		Xiamen Medical College Affiliated Haicang Hospital
		Quanzhou Children's Hospital of Fujian Province
		The First Affiliated Hospital of Fujian Medical University
		Zhongshan Hospital, Xiamen University
Guangdong	4	The Third Affiliated Hospital, Sun Yat‐sen University
		The First People's Hospital of Foshan
		Guangzhou First People's Hospital
		Shenzhen Children's Hospital
Hunan	1	Xiangya Hospital Central South University
Jilin	1	The First Hospital of Jilin University
Heilongjiang	1	The Second Affiliated Hospital of Harbin Medical University
Chongqing	2	Chongqing University Three Gorges Hospital
		Children's Hospital of Chongqing Medical University
Sichuan	2	West China Second University Hospital, Sichuan University
		Chengdu Women's and Children's Central Hospital
Shaanxi	1	Xi'an Children's Hospital
Ningxia	1	General Hospital of Ningxia Medical University

### 2.2 Ethics

#### 2.2.1 Independent ethics committee

The study protocol, case report form, informed consent form (ICF), and other study‐related information were reviewed and approved by the independent ethics committee (IEC) or institutional review board at each participating site before the study initiation.

#### 2.2.2 Code of ethics

This study was conducted in accordance with the Declaration of Helsinki and Good Clinical Practice. Prior to the initiation of the clinical study, the clinical study protocol, case report form, and ICF were reviewed and approved or filed by the IEC. The ICF, which was intended to protect the rights, safety, and well‐being of the participating children and approved by the IEC, was signed by each patient or his/her legal representative and the investigator, with the signed original copy kept by the investigator and a copy given to the patient or his/her legal representative. The inclusion and exclusion criteria were strictly formulated according to the code of medical ethics.

#### 2.2.3 Funding, investigators, and institutions

The study was sponsored by Beijing Hanmi Pharmaceutical Co., Ltd. The principal investigators were Professor Enmei Liu and Professor Quan Lu. The leading sites were Children's Hospital of Chongqing Medical University and Children's Hospital of Shanghai.

### 2.3 Database creation and data processing

#### 2.3.1 Target population

The inclusion criteria for participants were as follows: age ≤14 years; lower respiratory tract infectious diseases, including acute bronchitis, pneumonia, bronchial asthma with infection, and acute infectious bronchiolitis; and voluntary study participation with the ICF signed by patient's legal guardian or legally authorized representative. Children who could not provide a signed ICF were excluded from this study.

#### 2.3.2 Data extraction and download methods

To access the data from the database, investigators were required to submit a formal application to the project team and sign a data use agreement. They were allowed to access the database and retrieve relevant data after the application was approved.

### 2.4 Data content

**FIGURE 2:**
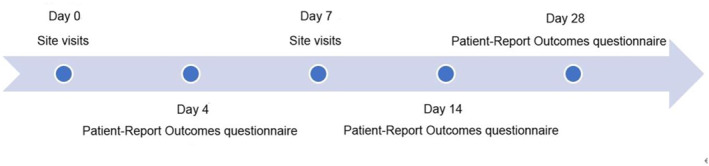
Study flowchart.

**FIGURE 3:**
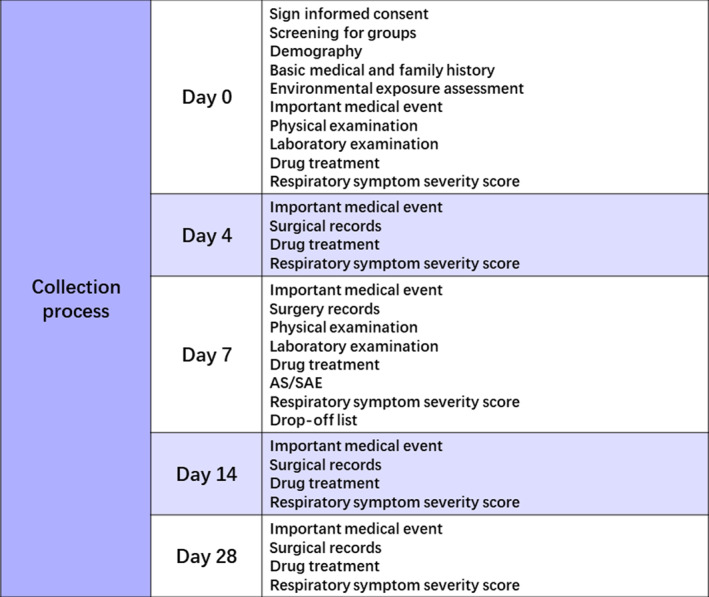
Times and specific contents of the data collected in the study.

The same pediatric respiratory specialists with prescription rights conducted on‐site visits on day 0 (baseline) and day 7, recorded the corresponding clinical data of the children, and collected information on the patient outcomes on days 4, 14, and 28 using the patient‐reported outcome questionnaire (see Table 2 for details on the types of data collected). The times and specific contents of the data collected in this study are shown below (Figures 2 and 3).

**TABLE 2:** Basic information for children with lower respiratory tract infection.

Variable	Number (*N*, %)
Source	
Outpatient	1088 (24.44)
Inpatient	3364 (75.56)
Sex	
Male	2636 (59.21)
Female	1816 (40.79)
Age	
0–28 days	12 (0.27)
28 days‐3 months	299 (6.72)
3–6 months	310 (6.96)
6 months‐1 year	597 (13.41)
1–3 years	1219 (27.38)
3–6 years	1269 (28.50)
6–14 years	746 (16.76)
Disease	
Acute bronchitis	922 (20.71)
Pneumonia	3225 (72.44)
Bronchial asthma with infection	171 (3.84)
Acute infectious bronchiolitis	52 (1.17)
Others	82 (1.84)
Treatment patterns	
Pathogen‐specific treatment	3305 (79.33)
Symptomatic treatment	3732 (89.58)
Adjuvant treatment	599 (14.38)
Chinese traditional medicine	1358 (32.6)
Other	376 (9.03)

The patient data collected during the on‐site visits included the following:

Demographics: date of birth, sex, ethnicity, health insurance type, guardian information (age, education level, occupation, etc.), and awareness of respiratory diseases.
Medical history: history of respiratory diseases, allergies (airborne, foodborne, drug‐induced, etc.), other chronic diseases, and family history of relevant conditions.
Anthropometric and vital data: measurements such as height, weight, heart rate, respiratory rate, body temperature, and systolic/diastolic blood pressure.
Environmental exposure: information regarding air (seasonal) and secondhand smoke exposure.
Disease characteristics: data related to disease type, disease severity, and related respiratory symptoms.
Drug treatment: details regarding the drug name, start time, dose, and any reasons for change in the treatment regimen.
Major medical events/complications: respiratory failure, heart failure, toxic encephalopathy, other severe diseases of the respiratory, cardiovascular, cerebrovascular, and digestive systems, and related medical outcomes.
Visual analog scale (VAS) scores: severity of clinical respiratory symptoms (cough, expectoration, and sputum sounds) and their impact on the quality of life of the children were assessed using VAS scores.
Laboratory data: relevant laboratory data were collected, such as complete blood count, C‐reactive protein level.
Adverse event: any adverse events experienced by the patients during the study were recorded.
Treatment compliance: the extent to that the patient completed the prescribed medication regimen was documented, along with specific reasons if the medication could not be taken according to medical advice.

### 2.5 Data quality and integrity

To ensure the quality and integrity of data, the investigators who conducted the on‐site visits were pediatric respiratory specialists with prescription rights, and the on‐site visits on days 0 and 7 were conducted by the same physician to reduce investigator‐introduced systematic errors in the data generated during the treatment of the same child. The status of any patient who did not show up for on‐site follow‐up was obtained via telephone follow‐up to ensure the integrity of data. VAS was used to assess patients' quality of life. The VAS score was chosen by patients after explanation of the VAS by the medical staff, or by their parent, legal guardian, or caregiver, if they were too young or could not adequately express themselves. In either case, VAS scoring must be completed by the same person at both baseline and follow‐up visits. The VAS scores at baseline and days 0, 4, 7, 14, and 28 were recorded.

### 2.6 Data analysis plan

All statistical analyses were performed using the SAS 9.4 software. Continuous variables (quantitative indicators) were statistically described using the number of cases, mean, standard deviation, median, interquartile range, minimum, and maximum. Categorical/ordinal variables (qualitative indicators) were statistically described using the frequency and percentage of each category or grade. The quantitative and qualitative indicators were compared across groups using *t*‐test, one‐way analysis of variance, Wilcoxon rank sum test, chi‐squared test, and Fisher exact test. Relevant factors affecting the improvements of respiratory symptoms in children were also analyzed.

## 3 INTRODUCTION TO EXISTING DATA

### 3.1 Research and analysis of the database's 2018–2019 operational data

The screening process for the first child with LRTI started on 23 May 2018. As of 31 July 2019, a total of 4855 children with LRTIs underwent the screening process. Among them, 4805 children from 36 medical centers were included in the study. Altogether, 4452 patients were evaluated for efficacy at least once after the baseline assessment and included in the full analysis set (FAS).

**FIGURE 4:**
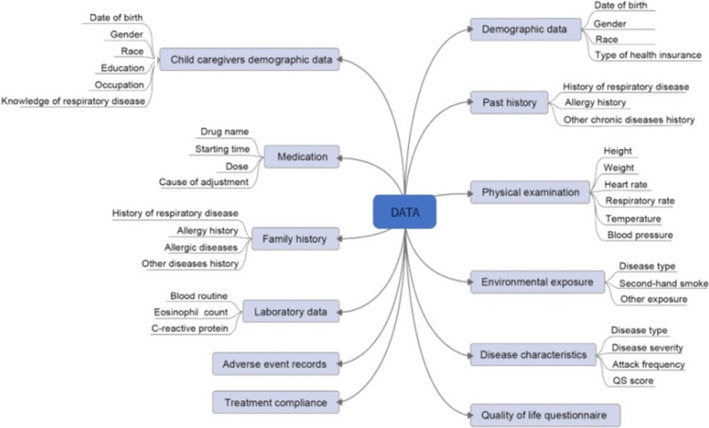
Clinical data collected from included patients.

The enrolled children received routine treatment (e.g., dietary adjustment, exercise, or medication). Corresponding data generated during routine clinical practice were accurately recorded to monitor improvements in the signs and symptoms of lower respiratory tract diseases in patients receiving drug treatment. To ensure accurate data collection, specific procedures for recording the corresponding data were implemented at each site (Figure 4).

### 3.2 Current database

Regarding patient distribution, a total of 3364 pediatric inpatients were included in the current database study, accounting for 75.56% of all patients included. On the other hand, 1088 pediatric outpatients were included, accounting for 24.44% of all patients.

With respect to sex, 2636 boys (59.21%) and 1816 girls (40.79%) were included in the FAS. In total, 2636 boys (59.21%) and 1816 girls (40.79%) were included in the FAS.

Regarding age, 2488 patients fell within the age range of 1–6 years, accounting for 55.88% of all patients. The mean age of the patients in the FAS was 3.26 ± 2.84 years, and the oldest patient had an age of 14.85 years.

Regarding disease type, pneumonia was the dominant disease type in the current database, accounting for 72.44% of the total cases, followed by acute bronchitis, which accounted for 20.71%.

Regarding treatment patterns, 4166 (93.58%) patients had medication records, while 286 patients (6.42%) did not. For the patients with medication records (*n* = 4,166, 93.58%), the treatment modalities were primarily categorized into pathogen‐specific treatment (*n* = 3,305, 79.33%) and symptomatic treatment (*n* = 3,732, 89.58%) (Table 2).

Regarding prognostic assessment, 4450 (99.96%) children with LRTIs had respiratory symptom scores (QS scores) rated at baseline, with a mean score of 5.08 ± 2.15. On day 7 after treatment, 4273 (95.98%) children had their QS scores rated again, with a mean score of 1.7 ± 1.56. Compared with baseline, the QS scores on day 7 after treatment were significantly lower (*p* < 0.001).

On day 7 after treatment, the proportion of children with moderate or more severe LRTI (*n* = 29, 0.65%) decreased significantly compared to baseline (*n* = 1,098, 24.66%) (*p* < 0.001). Furthermore, respiratory symptoms were clinically controlled in 46.11% of patients (*n* = 2053), and the total effective rate was 82.32%.

**FIGURE 5:**
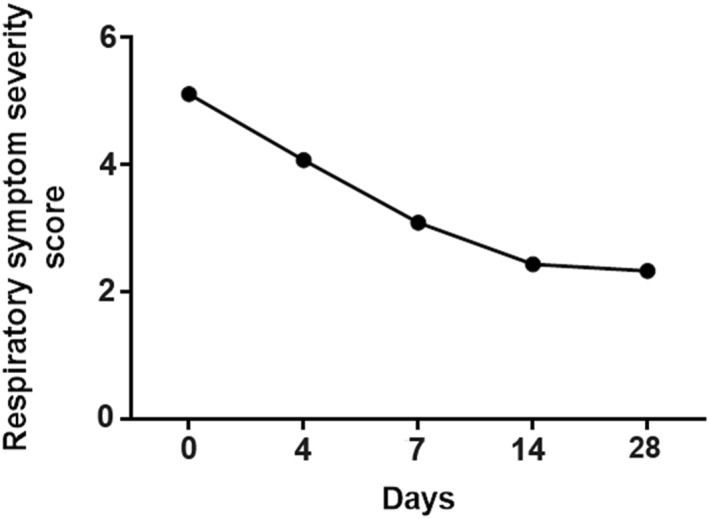
Respiratory symptom severity scores of patients after different treatment durations.

In the FAS, a total of 1853 (41.62%) patients with LRTIs had respiratory symptom severity (VAS score) rated by their guardians at baseline, with a mean VAS score of 5.11 ± 2.32. A total of 1924 (43.22%), 1644 (36.93%), 1423 (31.96%), and 1199 (26.93%) patients had VAS scores rated on day 4, day 7, day 14, and day 28 after treatment, respectively. The VAS scores decreased significantly over time (Figure 5).

With respect to the summary of adverse events, 14 patients (0.31% of the study population) experienced at least one adverse event. Among them, 12 had drug‐related adverse events, including two classified as moderate in severity. Additionally, 2 patients required drug replacement due to adverse events, and 10 discontinued the medication due to adverse events. No serious drug‐related adverse events were observed.

## 4 DISCUSSION

To date, only a few large‐scale, multicenter databases related to the treatment of LRTIs have been established worldwide. Most of the existing databases are collections of single‐center, single‐disease clinical data. Moreover, current studies in this area have primarily focused on anti‐infective treatments and adopted efficacy as the only outcome measure for comparison, which further limits their clinical significance. To the best of our knowledge, this study is the first national, noninterventional, observational, prospective, multicenter, real‐world study of LRTIs in Chinese children. This database enables observation of the efficacy of different treatment modalities, thus facilitating the integration of research findings into clinical practice for managing LRTIs in children. The results and data analysis from this study offer a straightforward, expeditious, and efficient method to store and manage essential information on the diagnosis, treatment modalities, and clinical data of LRTIs in Chinese children.

**FIGURE 6:**
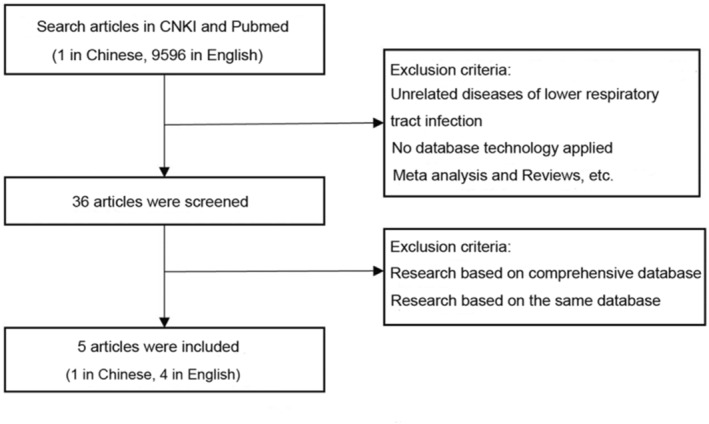
Literature screening flowchart.

By searching the literature (see Figure 6 for a flowchart of the literature screening process), we have identified the earliest database related to the treatment of LRTIs, that is, the respiratory syncytial virus (RSV) infection comprehensive database of the Personalization in Practice‐Networked Improvement Community established by Canadian pediatric researchers in 1993.^6^ In this database, medical personnel from nine hospitals participated in the collection of demographic information, daily clinical assessment data, oxygen saturation determination, and interventions (bronchodilators, steroids, ribavirin, antibiotics, intensive care, and mechanical ventilation) of children with RSV infection. This database was intended to explore the developmental trends of the disease under different interventions in children with RSV infection. Given its multicenter and large‐sample nature, the database offers valuable data for analysis. However, its early database structure and the simplicity of the collected data limit the extent of useful information that can be obtained from analysis, consequently restricting its clinical applicability.

With continuously increased understanding of lower respiratory tract infectious diseases, an increasing number of large‐scale, multicenter LRTI database studies have emerged, each focusing on specific research directions. Among these initiatives, TAVeM was the first international, multicenter, prospective, observational study focusing on ventilator‐associated LRITs, including pneumonia and tracheobronchitis.^7^ In the TAVeM study, 2960 eligible patients from 114 intensive care units in eight countries in Europe and South America (Spain, France, Portugal, Brazil, Argentina, Ecuador, Bolivia, and Colombia) were included. This database stores data pertaining to demographic characteristics, primary diagnosis, length of stay, McCabe classification of comorbidities, likelihood of survival, and prognosis, which were collected through electronic case report forms at a website for efficient data importing and management. This database allows for analyzing the associations between pneumonia or tracheobronchitis and ventilator‐associated LRTIs. However, it is essential to note that TAVeM focused only on ventilator‐associated LRTIs.

In 2018, the “TAILORED‐Treatment” study was jointly conducted by seven medical centers in the Netherlands and Israel. These centers developed a new clinical tool to distinguish viral infections from bacterial infections based on the collected demographics, medical history, clinical symptoms, physical examination, disease course, laboratory measurements, diagnosis, and telephone follow‐up data 28 days after the enrollment. The primary objective of this study was to develop new tools aimed at increasing the effectiveness and rationality of antibiotic treatment for LRTIs, reduce adverse events, and limit the emergence of antimicrobial resistance in children and adults.^8^ This database has complete baseline and follow‐up data and provides analysis and feedback on the response rates of treatment for LRTI in children and adults, thereby offering targeted and personalized treatment for patients with LRTIs. However, research utilizing this database is primarily focused on the anti‐infective treatment for LRTIs, and other treatment methods and modalities have not been involved extensively within the scope of this database.

During the literature search, we found only one database related to respiratory tract infections in children, namely the European Society for Pediatric and Neonatal Intensive Care Covid‐19 Pediatric and Neonatal Registry (EPICENTER). EPICENTER is a multicenter, multidisciplinary, metadata‐driven, hospital‐based, online, prospective cohort registry dedicated to neonatal and pediatric severe acute respiratory syndrome coronavirus 2 (SARS‐CoV‐2) infections.^9^ The database prospectively collects clinical data of hospitalized neonates and children infected by SARS‐CoV‐2, as well as that of neonates born to infected mothers. As of 3 May 2020, the registry has successfully enlisted approximately 100 centers worldwide. Each center collects demographics, clinical imaging, and laboratory data of children infected by SARS‐CoV‐2 to promote the diagnosis and treatment of coronavirus disease 2019 (COVID‐19) and the care of COVID‐19 patients. However, EPICENTER is confined to children infected by SARS‐CoV‐2, which narrows its application and limits its ability to address a broader range of respiratory tract infections in children.

Compared with the established database systems for LRTIs in other countries, the application of database systems for LRTIs in Chinese children is still in its infancy. During the severe acute respiratory syndrome (SARS) outbreak in 2002, Jiangsu Province established the Jiangsu Province SARS database to gather accurate data for analyzing the SARS epidemic, evaluating control measures and conducting research. This database comprises data collected through the disease prevention and control centers at all levels in Jiangsu Province and included information on clinically diagnosed cases, suspected cases, close contacts, and epidemiological survey results, providing strong support for the control of the SARS epidemic in this province.^10^ However, this database was specific to lower respiratory tract infectious diseases caused by SARS in Jiangsu Province, rendering it relevant only for a specific period and with a relatively limited sample size. Moreover, it was primarily intended for epidemic prevention rather than clinical research, which limited its value for scientific research. Although China has established a national, large‐scale, comprehensive database collecting data related to LRTIs, this database lacks specific data labels pertaining to LRTIs in children, complicating in‐depth data mining and resolving relevant clinical scientific inquiries.

In this study, clinical data of children with LRTIs were collected from 36 study sites in China through fixed patterns, such as on‐site visits or questionnaires at fixed time points, according to the timeline of patient visits. The database has several noteworthy characteristics: first, the database is open to all participating sites, and all the information in the database can be used to facilitate clinical research and academic exchange. Second, multiple types of data were collected, encompassing clinically relevant information, sociodemographic data of patients and their caregivers, treatment costs, benefits, adverse events, and complications. This database allows researchers and clinicians to explore differences in the efficacy and benefits of different treatment modalities for LRTIs and serves as a valuable data resource for conducting other case studies. Third, this national, multicenter, large‐sample database enables large‐scale data research, offering a comprehensive representation of LRTIs in children across regions and settings. Fourth, the data of each case were submitted by the same pediatric respiratory specialist with prescription rights. Accurate information ensures the authenticity of all conclusions and all published data based on the database system. Last, this database is the first national, large‐scale, multicenter, non‐interventional, real‐world prospective database of LRTIs in pediatric respiratory diseases in China.

With the rapid development of database technology, the data on single factors affecting a disease can be integrated into multifactor research projects. This integration enables the development of personalized medication and treatment modalities for patients with specific diseases, including LRTIs in children. Our next step is to build database‐related software and websites, and we will gradually standardize the data semantics and structure across the study sites to form a complete and sustainable data network. This network will not only facilitate further bioinformatics research, but also bridge the gap between clinical practice and the wealth of data available in the database, ultimately improving the outcomes and quality of care for children with LRTIs.

Our database is an essential resource for researchers and healthcare professionals. However, like any database, it has limitations. Here are some of the limitations and potential ways to address them: first, although this database includes cases from different provinces and cities in the east, south, west, and north of China that make it representative, it may not cover all regions, which limits the generalizability of the findings. To address this limitation, our team may collaborate to expand the database's coverage by including studies from underrepresented regions. This could involve establishing partnerships with research institutions in different regions or actively including studies from these regions in the database. Second, the database may not provide detailed information about the specific treatments used in the studies, making it difficult to assess the efficacy of individual treatment modalities. To address this limitation, researchers may enhance the database by including more treatment details, such as dosage, duration, and specific medications. This could be done by re‐reviewing the original studies and extracting additional relevant data or by including a more detailed standardized template for reporting treatment information in future studies. Third, the collection of data for this database was stopped before the outbreak of COVID‐19. This pandemic has caused significant impact on all aspects, resulting in suspension of domestic data collection. The restart of case collection for the database is now in the planning phase. Regular update of the database with new findings and treatment protocols would keep it in pace with evolving medical knowledge. Overall, addressing these limitations requires collaboration among researchers, healthcare professionals, and other stakeholders. By expanding the coverage of the database, enhancing the details of treatment information, and distinguishing between different types of infections, the database can provide more comprehensive and actionable insights into the efficacy of different treatment modalities for LRTIs in children.

## AUTHOR CONTRIBUTIONS

Dr Deng conceived and designed the work, carried out the initial analyses, drafted the initial manuscript, and revised the manuscript. Prof Liu and Prof Lu conceived and designed the work, and revised the manuscript. Zengni Li, Zhengxiu Luo, Guangli Zhang, Bing Hu, Yingxue Zou, Jinhai Ma, Changshan Liu, Xiaoyan Dong, Huifen Zi, Chuangli Hao, Rongjun Lin, Xiangrong Zheng, Bingfei Li, Fenhua Chen, Mei Fang, Weimin Tian, Zhiqiang Zhuo, Deyu Zhao, Zhimin Chen, Yuejie Zheng, Jingyang Zheng, Yong Yin, Qiuyu Tang, Liqun Wu, Li Gu, Jinzhun Wu, Liyi He, Tao Ai, Ning Wang, Minjun Zhang, Hailin Zhang, Hanmin Liu, Youxiang Zhang, Jianguo Hong, Zhiying Han, Yunbo Mo, Hongmei Qiao and Zhiliang Tian acquired the medical records, analyzed data for the work, and revised the manuscript. Yong Yin involved in Investigation, writing – review and editing of the manuscript.

## CONFLICT OF INTEREST STATEMENT

The authors declare that they have no conflict of interest.

## ETHICS STATEMENT

Ethical approval for this study was obtained from the Institutional Review Board of the Children's Hospital of Chongqing Medical University (Ethics Committee Approval No. 2018‐2). In addition, this study has been registered at the Chinese Clinical Trial Registry (Registration No. ChiCTR1800015818). The study protocol, case report form, informed consent form (ICF), and other study‐related information were reviewed and approved by the independent ethics committee (IEC) or institutional review board at each participating site before the study initiation.

## PATIENT CONSENT STATEMENT

The ICF, which was intended to protect the rights, safety, and well‐being of the participating children and approved by the IEC, was signed by each patient or his/her legal representative and the investigator, with the signed original copy kept by the investigator and a copy given to the patient or his/her legal representative.

## PERMISSION TO REPRODUCE MATERIAL FROM OTHER SOURCES

Not applicable.

## CLINICAL TRIAL REGISTRATION

Chinese Clinical Trial Registry [ChiCTR1800015818].

## CONSENT FOR PUBLICATION

Not applicable.

## Data Availability

Data supporting the findings of this study are available on request.
